# Editorial: Aldehyde dehydrogenase in clinical settings: Potential biomarker and therapeutic target in solid tumors

**DOI:** 10.3389/fmed.2022.1116908

**Published:** 2023-01-04

**Authors:** Beatrice Aramini, Valentina Masciale

**Affiliations:** ^1^Division of Thoracic Surgery, Department of Experimental, Diagnostic and Specialty Medicine—DIMES of the Alma Mater Studiorum, University of Bologna, G.B. Morgagni—L. Pierantoni Hospital, Forlí, Italy; ^2^Division of Oncology, Department of Medical and Surgical Sciences, University of Modena and Reggio Emilia, Modena, Italy

**Keywords:** aldehyde dehydrogenase (ALDH), cancer stem cell (CSC), NSCLC, recurrence, nanoparticle

In humans, the acetaldehyde dehydrogenase (ALDH) family contains 19 active isoenzymes that are expressed in the majority of mammalian tissues [Zanoni et al.; (1)]. Each ALDH isoform exhibits a specific expression pattern and has an independent functional role in cancer (2, 3). The ALDH1A1 isoform detoxifies the enzymes that catalyze the oxidation of intracellular aldehydes (4). Clinical studies have shown that this isoenzyme is highly expressed in several solid tumors, such as lung cancer (4). Additionally, previous work has demonstrated that ALDHs are overexpressed in cancer stem cells (CSCs), which are subpopulations of cancer cells with stem-like features such as unlimited proliferative potential and drug resistance (5). These features both represent major clinical challenges in many cancer types. ALDH1A1 has been recently described as a marker for identifying and isolating human CSCs in non-small cell lung cancer (6, 7). Moreover, ALDH1A1 is involved in several cellular processes such as differentiation, retinoic acid (RA) synthesis, and the detoxification and regulation of amino acid and lipid metabolism (8). Specifically, ALDH enzymes enable cancer cells to metabolize toxic aldehydes into carboxylic acids, which are less reactive and more soluble. This function is particularly advantageous in the setting of anti-tumor therapies that facilitate aldehyde accumulation and DNA double-strand break formation *via* lipid peroxidation and reactive oxygen species (ROS) generation (Zanoni et al.).

In addition to promoting CSCs survival, ALDH overexpression can also influence immune cells. Specifically, RA signaling-mediated reductions in ROS impair immunogenic cell death by activating and stabilizing immunosuppressive regulatory T cells (Tregs) [Zanoni et al.; (9, 10)]. In particular, Tregs are crucial for promoting immune tolerance and preventing aberrant immune responses. On the other hand, they compromise anti-tumor immunity and promote the progression of many different carcinoma types. This phenomenon is particularly evident in tumors of the gastrointestinal tract, in which high levels of ALDH1A1 are associated with worse patient prognosis (10). Moreover, previous studies have shown that increased ALDH1 expression positively correlates with resistance to radiation therapy and chemotherapy, as well as with malignant progression, in cancer patients (10, 11). This relationship is due to the capacity of ALDH1A to modulate the intracellular pH within tumors, as well as the activation of drug-resistance pathways such as the USP28/MYC, ALDH1A1/HIF-1α/VEGF, and Wnt/β-catenin axes (Wei et al.). Therefore, ongoing research is exploring ALDH1 as a therapeutic target in several solid tumors. Inhibition of ALDH1 may reduce cancer cell proliferation, thereby suppressing the recurrence and metastasis of malignant tumors (Wei et al.).

Despite several recent studies seeking to characterize ALDH inhibitors for the treatment of solid tumors, there is no clinical evidence available that attests to their efficacy or safety, potentially due to their high toxicity and limited efficacy and/or bioavailability [Zanoni et al.; (9–11); Wei et al.]. Moreover, many of these inhibitors have been effective in preclinical models of solid tumors. Therefore, a deeper understanding of ALDHs as therapeutic targets will be crucial for the development of anti-neoplastic ALDH inhibitors. In particular, it may be important to focus on protein domains other than the catalytic domain, such as isoform-specific domains (12–14). However, the paucity of crystallographic structural data and isoenzyme-specific assays has limited the development of novel effective and specific inhibitors (15–17). Nevertheless, the isoform-specific inhibitors that are available are effective when combined with other therapies but show limited efficacy when used as monotherapies, ostensibly due to the compensatory upregulation of other ALDH enzymes (18). Thus, the use of multi-isoform ALDH inhibitors may represent a more powerful strategy. In addition, multi-ALDH inhibitors synergize with conventional treatments such as chemotherapy, targeted therapies, and radiotherapy, to inhibit disease progression and prevent the onset of resistance (18, 19). Furthermore, several strategies to improve bioavailability and reduce toxicity, such as novel nano-formulations and drug-delivery devices, are being analyzed in preclinical models. These approaches have been shown to reduce the incidence of off-target effects by improving both biodistribution and pharmacokinetics (20). Furthermore, ALDH inhibition may improve the ratio of effector T cells to Tregs within tumor tissue, leading to enhanced antitumor immunity, while simultaneously impacting ALDH-expressing cancer cells (10, 11). This intriguing approach may be particularly efficacious when combined with immunotherapy. Specifically, the inhibition of ALDH activity and ensuing reductions in RA availability in the tumor microenvironment (TME) may negatively influence Treg cell differentiation and activation, attenuating their immunosuppressive functions (21). Coupling ALDH inhibitors with PD-1 or PD-L1 blockade, for example, may also restore the activity of exhausted CD8^+^ effector T cells, thereby activating anti-tumor immunity. Therefore, dissecting the interactions between the myriad ALDH1A1-overexpressing cell types in the TME is critical for maximizing the efficacy of combinatorial approaches, enabling the inhibition of pro-tumor Tregs, and enhancing T cell-mediated tumor eradication (10, 11, 21–23).

In summary, ALDH inhibitors may synergize with several conventional therapies to both inhibit disease progression and prevent the development of resistance (24). Given that combinations of standard treatments often represent the standard of care for cancer patients, the identification of novel complementary approaches, including those that incorporate ALDH inhibitors, is required to overcome recurrence and drug resistance.

## Author contributions

BA wrote and revised the editorial. VM revised the editorial. Both authors contributed to the article and approved the submitted version.
